# The efficacy of surgery over stereotactic radiosurgery in the management of tumor-related trigeminal neuralgia

**DOI:** 10.1186/s41016-024-00379-y

**Published:** 2024-10-01

**Authors:** Alexander Abouharb, Hasithe Rathnayake, Sachit Mehta

**Affiliations:** 1 School of Medicine Worsley Building, University of Leeds, Woodhouse, Leeds, LS2 9JT UK; 2 GKT School of Medical Education, King’s College London, Strand, London, WC2R 2LS UK; 3 Faculty of Medicine, St Mary’s Hospital, Imperial College London, Praed St, London, W2 1NY UK

**Keywords:** Trigeminal neuralgia, Surgery, Stereotactic radiosurgery, Tumors

## Abstract

Tumor-related trigeminal neuralgia (TN) is a deeply debilitating condition that severely impacts patient quality of life. Two principal treatment methods in use are open surgical resection of the causative tumor or the use of stereotactic radiosurgery (SRS). In this letter, we aim to evaluate the use of both treatment methods and highlight that in patients with commensurate anatomy, open surgical resection continues to provide greater rates of symptomatic relief, lower rates of recurrence, and complication compared to stereotactic radiosurgery.

Dear Editor,

Trigeminal neuralgia (TN) is a debilitating condition, defined by chronic pain across the dermatome innervated by the trigeminal nerve. The pathophysiology of TN is multifaceted, though typically occurs due to trigeminal compression, resulting in severe neuropathic pain. Several mechanisms may cause trigeminal compression, allowing TN to be grouped into classic TN, secondary TN, and idiopathic TN, as per the International Headache Society [1]. Of these, perhaps the most treatable form is secondary TN, in which compression is caused by an underlying disease, commonly a neoplasm [1]. Several tumor types have been implicated, though it most often occurs secondary to trigeminal schwannomas, petroclival meningiomas, and cerebellopontine angle tumors [2]. Several treatment methods have been posited for secondary TN, though open surgery or stereotactic radiosurgery (SRS) is the mainstays of treatment. Here, we will evaluate the utility of these two treatment methods and highlight the continued relevance of open surgical resection in the management of this challenging and debilitating condition.

Cerebellopontine angle tumors are commonly causative for secondary TN. Their treatment with open surgical resection against SRS has been evaluated by several recent studies. The findings of Neff et al., who compared outcomes of both procedures to treat patients with large, vestibular schwannomas, are of particular interest. In their retrospective study comparing outcomes at 30-month post-index procedure, they found that for patients receiving surgical resection, 58% reported improvements in symptoms of neuralgia and paresthesia, compared to 0% of patients undergoing SRS reporting improvement in these symptoms post-treatment (*p* < 0.05) [3]. Other researchers have also documented the risks associated with increased trigeminal pain following SRS compared to open surgical resection. In their study evaluating pain following SRS for the treatment of schwannomas and meningiomas, Park et al. found that half of patients treated for TN secondary to meningiomas had worsened symptoms following SRS [4].

The findings of Nugroho et al. are also striking. In their systematic review evaluating open surgery versus stereotactic radiosurgery in the treatment of tumor-related TN, 26 retrospective studies were compared with Nugroho et al. finding that in patients undergoing open surgery, 92.2% gained pain improvement, 2.8% were unchanged, and 4.5% had recurrence, though none of the patients had worsened outcomes [2]. In cases treated with r-targeted radiosurgery, the improvement rate was 79.1%, unchanged at 14.3%, recurrence at 26.5%, and worse symptoms rate after the intervention was 6.6% [2]. The contemporary findings of Nugroho et al. highlight that in patients with tumors appropriate for surgical resection, greater symptomatic improvement may be anticipated, with rates of recurrence comparable or lower than radiosurgery.

Tumor-related TN can be caused by a range of intracranial tumors. They can be grouped depending on whether they compress or encase the trigeminal nerve [2], which impacts on the preferred treatment method and how appropriate they are for surgical resection. Prior research has shown that for isolated, solitary tumors, surgery confers improved survival rates, though carries a greater risk of iatrogenic injury [5]. For complex tumors, like those encasing the trigeminal nerve, SRS, a noninvasive procedure, may pose a lower risk of iatrogenic injury. However, as observed by some researchers, SRS, while less invasive, carries a greater risk of symptoms worsening than surgical resection [2]. Several reasons have been postulated for the worsened trigeminal symptoms in patients who have undergone SRS. Typically, in patients treated with SRS, tumor size does not decrease, and, instead, patients often experience temporary enlargement of their tumor, which causes greater trigeminal compression and only exacerbates their trigeminal symptoms [6]. In addition, exposure of the trigeminal nerve to radiation, as occurs in SRS, can cause permanent damage trigeminal nerve fibers, potentially causing permanent worsening of trigeminal symptoms [7, 8].

Despite the limitations of stereotactic radiosurgery, there are several situations in which this treatment method is preferential to open surgical resection. Surgical resection is associated with an increased rate of postoperative facial hypesthesia, due to iatrogenic injury to the trigeminal nerve intraoperatively [2], and this complication is unique to surgical resection, and in instances where tumor anatomy makes trigeminal nerve damage due to iatrogenic injury likely, SRS may be a superior treatment method. Radiosurgery is well documented as the least invasive surgical procedure for the treatment of trigeminal neuralgia and has been associated with relatively low rates of facial paresthesia’s after treatment and low recurrence in patients achieving full symptomatic relief [9]. Tumor size, overall patient health, and patient preferences are also factors that should be considered when deciding on surgery vs SRS. Generally, rapidly growing tumors, and/or those that precipitate edema, and those with a large diameter (> 4 cm) merit surgery, while patients who are highly comorbid and at high risk of perioperative complications may benefit from SRS [10, 11]. Each procedure carries its own benefits and risks, as outlined; therefore, the preferences of individual patients must be considered and always respected when feasible.

Despite some advantages of SRS, open surgical resection, when chosen to treat patients with appropriate tumor anatomy, and in the hands of experienced surgeons, is associated with improved rates of symptomatic relief, lower rates of complication, and lower rates of symptomatic recurrence, on average, and should continue to be the treatment of choice in patients suffering from tumor-related trigeminal neuralgia.

## Data Availability

No data was used in the preparation of this manuscript.

## Abbreviations

TN: Trigeminal neuralgia
SRS: Stereotactic radiosurgery
